# Overlap of Toxic Epidermal Necrolysis (TEN)-Like Cutaneous Lupus and Rowell Syndrome in Systemic Lupus Erythematosus: A Case-Based Review

**DOI:** 10.7759/cureus.95603

**Published:** 2025-10-28

**Authors:** Raja A Bakhsh, Kholood A Almehmadi, Homaid Alotaibi, Abdulrahman Saleh Aldairi, Faris Alsaedi, Sarab S Alharthi, Hanan O Alkaabi, Bashaer S Khawandanah, Hanin H Alsharif, Raghad T Alharbi, Sharaf M Alsharif, Wejdan M Alzahrani, Khaled S ِAldairi

**Affiliations:** 1 Department of Internal Medicine, King Faisal Hospital, Ministry of Health, Makkah, SAU; 2 Department of Dermatology, King Faisal Hospital, Ministry of Health, Makkah, SAU; 3 College of Medicine, Umm Al-Qura University, Makkah, SAU; 4 Department of Internal Medicine, Division of Rheumatology, King Faisal Hospital, Ministry of Health, Makkah, SAU

**Keywords:** case-based review, drug-induced ten, rowell syndrome, systemic lupus erythematosus, ten-like acute cutaneous lupus erythematosus

## Abstract

Systemic lupus erythematosus (SLE) is a chronic autoimmune disease with a broad range of systemic and cutaneous manifestations. Among its rare dermatologic variants are toxic epidermal necrolysis (TEN)-like acute cutaneous lupus erythematosus (ACLE), marked by erythema multiforme-like lesions and a distinct immunologic profile, and Rowell syndrome (RS). Unlike drug-induced TEN, both are autoimmune conditions. In this patient, the two entities did not coexist, but their striking clinical resemblance posed a significant diagnostic challenge. A 49-year-old Indonesian woman with a previously unremarkable medical history presented with a diffuse erythematous eruption beginning on the face and then spreading downward to involve the trunk and extremities, mucocutaneous involvement, and reticulated purpura. Laboratory investigations revealed severe hyponatremia, renal impairment, anemia, thrombocytopenia, low complement levels, ANA, anti-dsDNA, anti-Sm, anti-Ro/SSA, and anti-La/SSB antibodies, and all had strongly positive serology. Skin biopsy findings included full-thickness epidermal necrosis with interface dermatitis, thereby confirming the diagnosis of TEN-like ACLE. The patient was treated with systemic immunosuppression with agents including high-dose corticosteroids and mycophenolate mofetil together with supportive care, resulting in clinical stabilization. This uncommon and diagnostically challenging cutaneous manifestation of SLE carries significant clinical implications, particularly when distinguishing between TEN-like ACLE, RS, and drug-induced TEN. Prompt identification and timely initiation of immunosuppressive therapy are critical to reducing morbidity and preventing potential mortality. Additionally, we provide a comprehensive review of previously reported cases and discuss current diagnostic strategies and therapeutic approaches.

## Introduction

Systemic lupus erythematosus (SLE) is a chronic autoimmune disease characterized by multisystem involvement and a variety of dermatologic manifestations. Nearly 70-80% of patients with SLE get cutaneous lesions during the course of the disease, which include different forms of cutaneous lupus erythematosus (CLE) like acute, subacute, and chronic variants [1]. Among these, toxic epidermal necrolysis (TEN)-like acute cutaneous lupus erythematosus (ACLE) is one of the extremely rare and severe forms that mimic drug-induced TEN clinically but develop as an autoimmune phenomenon rather than a hypersensitivity reaction [2,3]. TEN-like ACLE presents with extensive epidermal detachment, necrosis, and mucocutaneous involvement; hence, procedures differentiating it from drug-induced TEN are very much necessary owing to the differences in its pathogenesis as well as management [4,5].

Another rare situation found in association with lupus erythematosus is Rowell syndrome (RS), originally explained by Rowell et al. in 1963 as the co-existence of lupus erythematosus (systemic or cutaneous) with erythema multiforme-like lesions with a distinctive immunologic pattern, including positive antinuclear antibody (ANA) and anti-Ro/SSA or anti-La/SSB antibodies [6,7].

RS diagnostic criteria have evolved over time, but its status as a distinct entity is still controversial, with many authors considering it a variant of subacute CLE [6]. Recognition of RS remains important owing to the great overlap with other blistering and targetoid dermatoses, chiefly erythema multiforme and drug eruptions [6].

We report a case of a middle-aged woman with SLE who presented with TEN-like ACLE that clinically mimicked RS but did not fulfill the diagnostic criteria of RS. This case highlights the diagnostic challenge posed by overlapping lupus variants and underscores the critical need to distinguish them from drug-induced TEN to ensure timely and appropriate therapy. In doing so, this report aims to clearly delineate the clinical and immunologic boundaries between TEN-like ACLE and RS, emphasizing their diagnostic and therapeutic implications. By reviewing previously reported cases, it also contributes to the growing understanding of these rare lupus variants, helping clinicians recognize and manage similar presentations more effectively.

## Case presentation

Patient information and history of the present illness

A 49-year-old Indonesian woman, with no previously known chronic medical conditions, presented to the emergency department with a one-year history of recurrent facial rash, which had worsened significantly over the preceding five days, becoming diffuse over the trunk and limbs bilaterally. Due to a language barrier, the history was primarily obtained through a friend accompanying her husband. Over the past year, the patient had multiple visits to private clinics for recurrent episodes of facial rash. She reportedly received various medications during these visits; however, her husband was unaware of the specific drugs prescribed or the working diagnoses. For the current episode, she had been taking oral fluconazole 150 mg once weekly for the past four weeks, though the indication for this treatment remained unclear.

The initial lesion appeared on the face and progressively spread to involve the lips, interfering with her ability to eat and speak, and subsequently extended to the neck, trunk, and all extremities, including the palms and soles. The patient also reported an undocumented fever prior to admission. Four days before the presentation, she developed bilateral foot pain.

There was no history of joint pain, morning stiffness, altered consciousness, convulsions, abdominal pain, nausea, vomiting, chest pain, dyspnea, cough, dysuria, or hematuria. However, one year earlier, the patient had experienced sore throat, shortness of breath, and vomiting, leading to admission at Makkah Medical Center, where she was discharged on allopurinol, levocetirizine, montelukast, and prednisone (tapered over one year). The reason for the prior hospitalization was unclear, though her husband recalled a mention of “kidney-related issues.” The patient denied any past surgical history, family history of chronic or autoimmune diseases, or recent infectious contacts. The indication for allopurinol use was not documented, and the medication had been discontinued for approximately one year before the current illness, making drug-induced TEN secondary to allopurinol highly unlikely.

Physical examination

On examination, the patient was conscious, alert, and fully oriented to time, place, and person (Glasgow Coma Scale score of 15/15). She appeared comfortable at rest but was noted to be shivering. Her vital signs were stable, with a blood pressure of 128/70 mmHg, a heart rate of 85 beats per minute, a respiratory rate of 20 breaths per minute, a body temperature of 36.9°C, and an oxygen saturation of 100% on room air.

**Figure 1:**
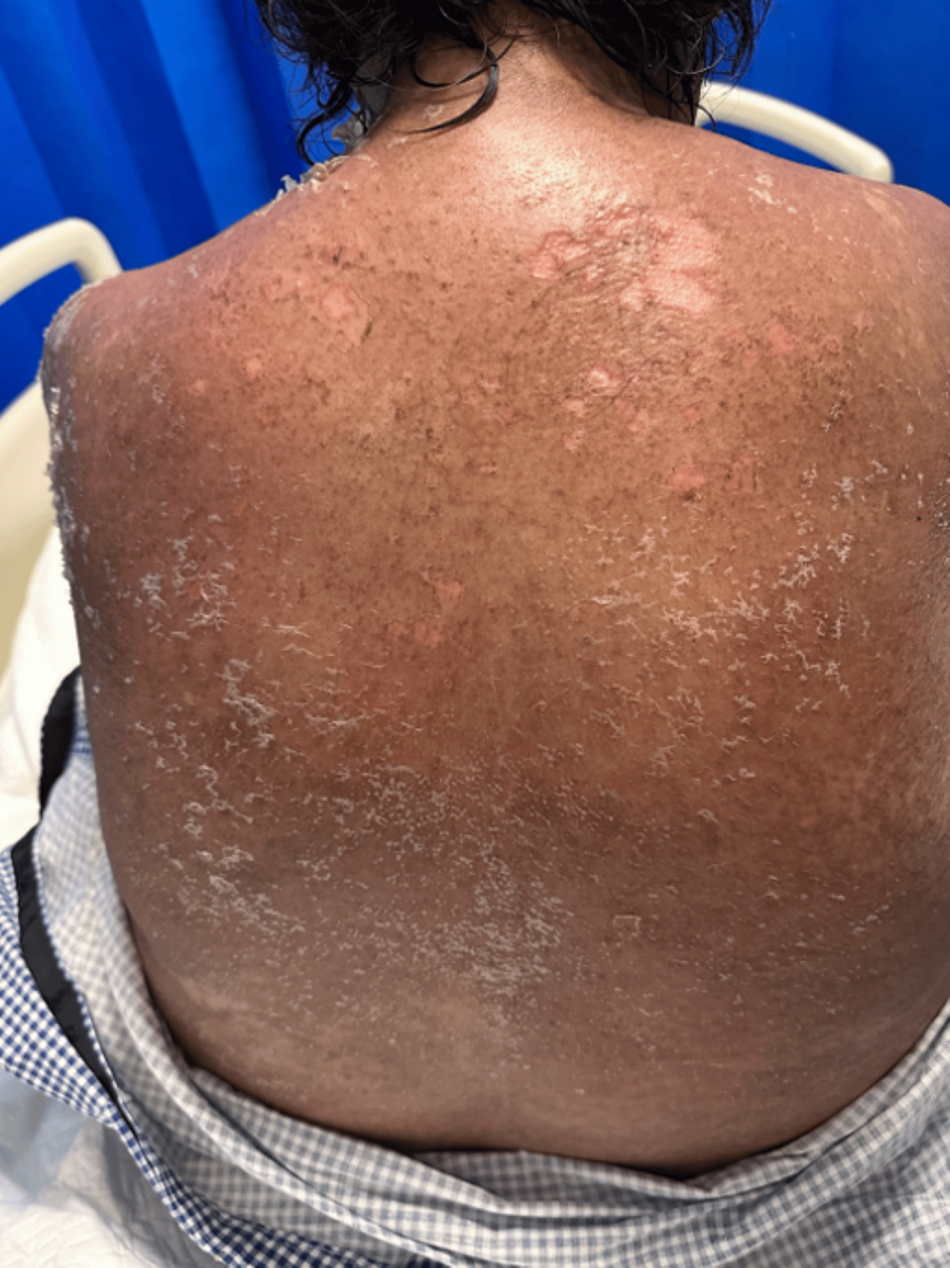
Diffuse erythematous plaques with fine to moderate scaling and peeling over the back and shoulders.

Dermatological examination revealed erythematous, edematous, non-tender plaques with areas of crusting and erosion distributed over the eyebrows, nasal bridge, perioral region, and malar areas, with sparing of the periorbital region. In the oral cavity, there was hemorrhagic crusting and erosion on the lips, while the oral mucosa itself remained unaffected. The anterior and posterior trunk shows diffuse erythematous plaques with overlying fine to moderate scaling. Some plaques are confluent with irregular borders, predominantly over the back and shoulders, with superficial epidermal peeling and focal areas of erosion noted (Figure 1). Examination of the abdomen and genital region showed a diffuse bright erythematous maculopapular rash, with early epidermal detachment in some areas and genital involvement localized to the pubic area.

**Figure 2:**
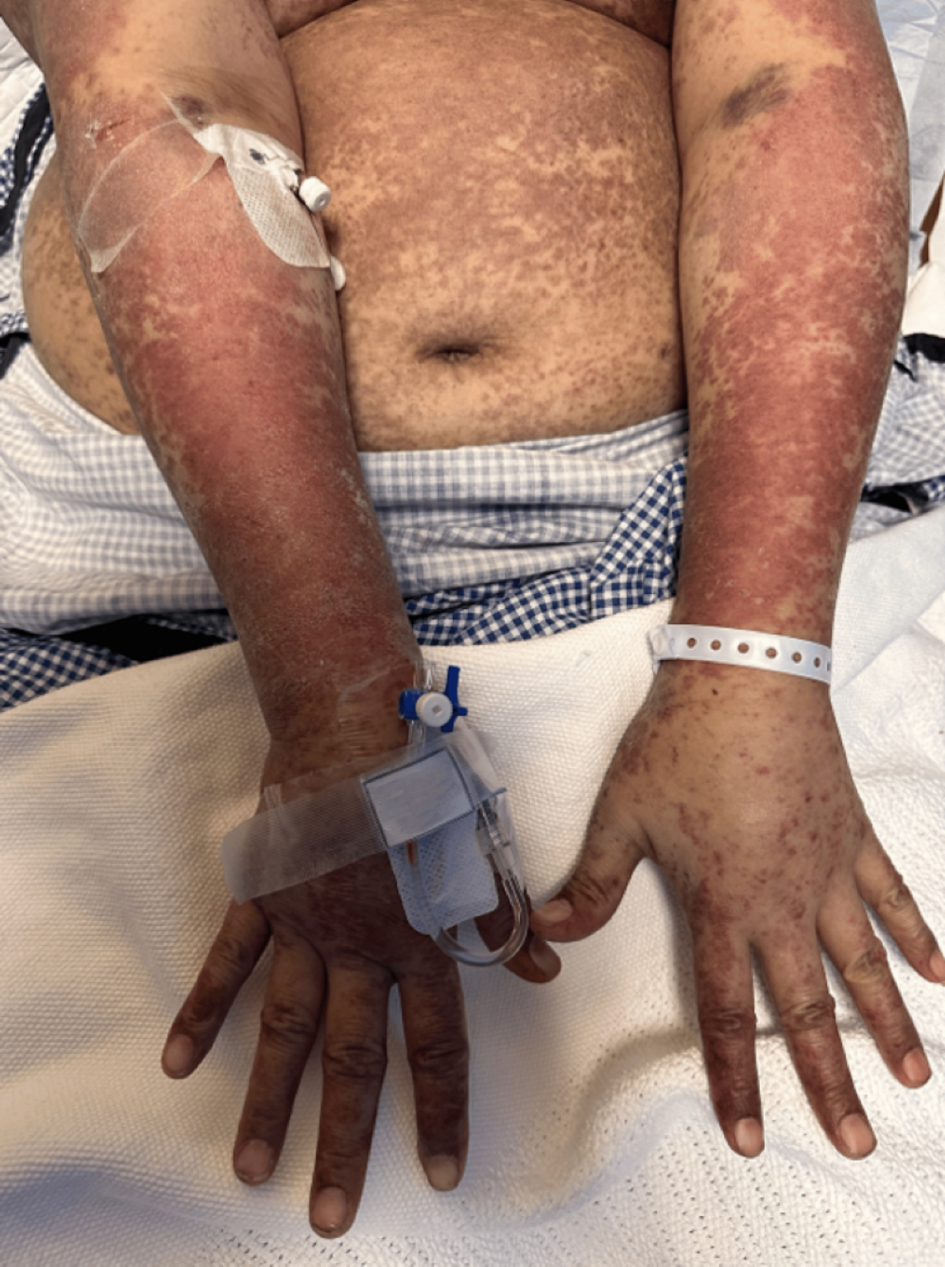
Diffuse erythematous to violaceous plaques with reticulated distribution involving both upper extremities, extending to the dorsal hands and fingers, with associated scaling and purpuric macules.

**Figure 3:**
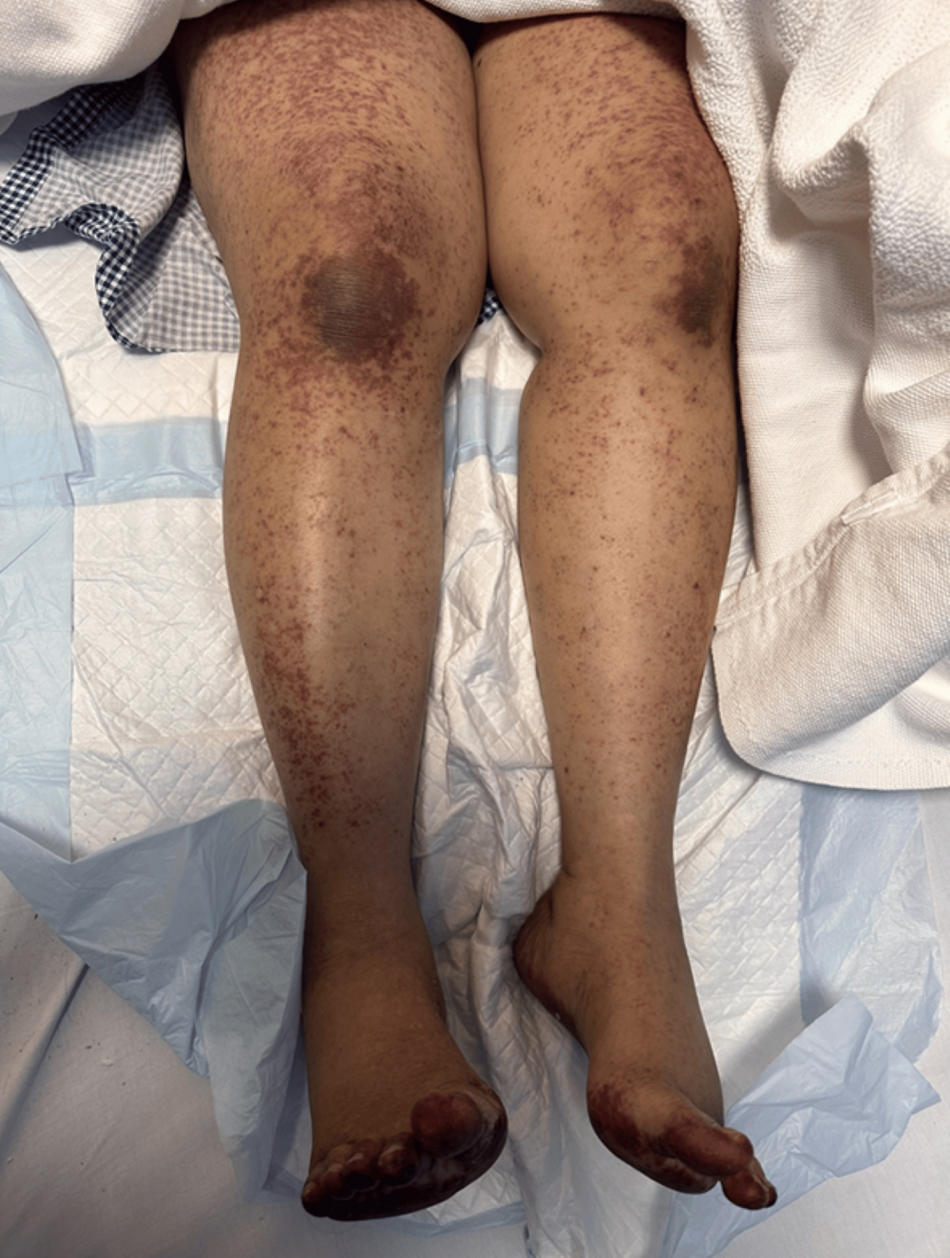
Non-blanchable purpuric macules in the lower limb.

**Figure 4:**
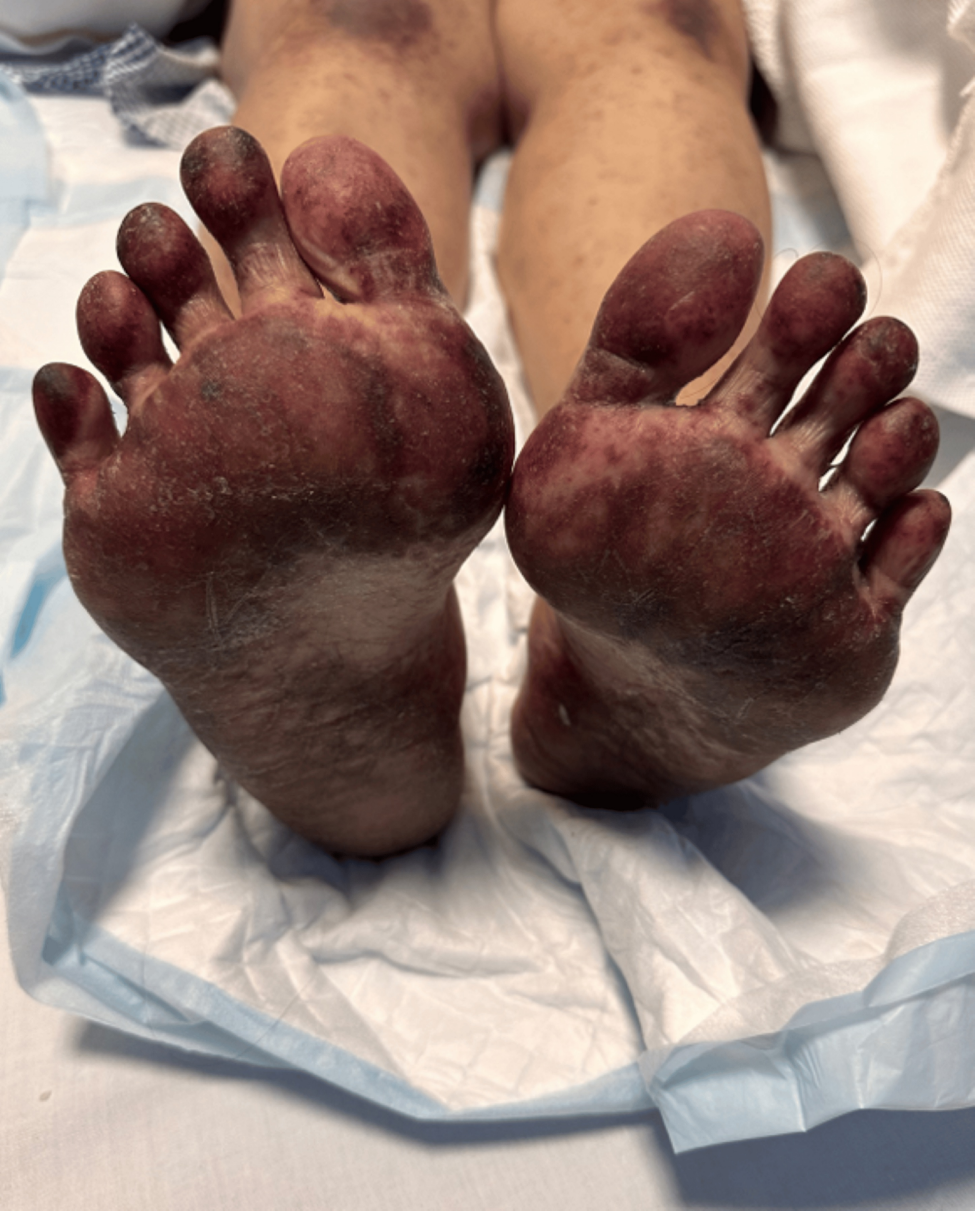
Dusky erythematous to violaceous plaques on the soles, with toes darkened to a deep violaceous hue with blisters covering the entire feet bilaterally, except for the medial arch (foot necrolysis).

On the extremities, the upper extremities show diffuse erythematous to violaceous plaques with a reticulated, confluent distribution. The lesions extend from the arms to the dorsal aspects of the hands and fingers. The skin surface shows areas of edema, scaling, and ill-defined erythematous patches intermixed with dusky discoloration. Over the hands and fingers, there is a more pronounced violaceous hue with scattered purpuric macules and erosions (Figure 2). While lower extremities show non-blanchable purpuric macular lesions extended from the thighs down to the feet (Figure 3), with marked plantar tenderness and dusky erythematous to violaceous plaques, the toes have darkened to a deep violaceous or purplish-red hue (Figure 4).

Laboratory investigations

Laboratory workup was performed to evaluate systemic involvement and possible autoimmune etiology. The results demonstrated hematologic abnormalities, impaired renal and hepatic function, evidence of active inflammation, and a strongly positive autoimmune profile consistent with SLE. Key findings are summarized in Table 1.

**Table 1:** Laboratory investigations showing multi-system abnormalities with autoimmune markers confirming the diagnosis of systemic lupus erythematosus. Detailed laboratory profile of the patient, revealing anemia, thrombocytopenia, renal impairment, hypoalbuminemia, electrolyte disturbances, hypocomplementemia, and strongly positive lupus serology, consistent with systemic lupus erythematosus activity. Symbols denote the intensity or presence of findings: “−” = absent or negative; “+” = mild or weakly positive; “++” = moderate; “+++” = marked; “++++” = very strong. WBC, white blood cell count; MCV, mean corpuscular volume; MCHC, mean corpuscular hemoglobin concentration; ESR, erythrocyte sedimentation rate; CRP, C-reactive protein; PT, prothrombin time; INR, international normalized ratio; AST, aspartate aminotransferase; ALT, alanine aminotransferase; ALP, alkaline phosphatase; ANA, antinuclear antibody; dsDNA, double-stranded DNA; Sm, Smith antigen; RNP, ribonucleoprotein; SS-A (Ro60), Sjögren’s syndrome antigen A; SS-B (La), Sjögren’s syndrome antigen B; C3, complement component 3; C4, complement component 4; RF, rheumatoid factor; IgM, immunoglobulin M; IgE, immunoglobulin E; PTH, parathyroid hormone; BUN, blood urea nitrogen.

Category	Parameter	Result	Reference Range/Interpretation
Complete Blood Count	WBC	4.12 ×10⁹/L	Normal
	Hemoglobin	109 g/L	Low
	MCV	80.9 fL	Low
	MCHC	368 g/L	High
	Platelets	119 ×10⁹/L	Low
	ESR	55 mm/h	High
	CRP	<0.5 mg/L	Normal
Coagulation Profile	PT	12.4 sec	Normal
	INR	0.91	Normal
Liver Function	AST	97 IU/L	Elevated
	ALT	39 IU/L	Slightly elevated
	ALP	59 IU/L	Normal
	Albumin	28.4 g/L	Low
	Total Bilirubin	5.8 μmol/L	Normal
Renal Function	Sodium	114 mmol/L	Low
	Potassium	4.76 mmol/L	Normal
	BUN	17.34 mmol/L	High
	Creatinine	187.6 μmol/L	High
Autoimmune Profile	ANA	Positive	Coarse/fine speckled pattern
	Anti-dsDNA	59.6 IU/mL	High
	Anti-Sm	+++	Positive
	Sm/RNP	+++	Positive
	SS-A (Ro60)	+++	Positive
	SS-B (La)	+	Positive
	Direct Coombs	Positive	Suggests autoimmune hemolysis; supports lupus-related immune-mediated red cell destruction.
	C3	<40 mg/dL	Low
	C4	<8 mg/dL	Low
	RF	<20 IU/mL	Normal
	IgM	284 mg/dL	High
	IgE	529 IU/mL	High
Urinalysis	Proteinuria	175.97 mg/dL	Mild (subnephrotic-range) proteinuria.
	24-hour urine protein	492.72 mg/day	Below nephrotic range.
	Urine culture	No growth	No evidence of urinary tract infection.
Other Tests	Calcium	1.5 mmol/L	Low
	Phosphorus	1.86 mmol/L	High
	PTH	25.6 pmol/L	High
	Cortisol	>123 μg/dL	High
	Ferritin	791 ng/mL	High
	Vitamin D	65.1 nmol/L	Low
	Uric acid	751.8 μmol/L	High

Histopathology

**Figure 5:**
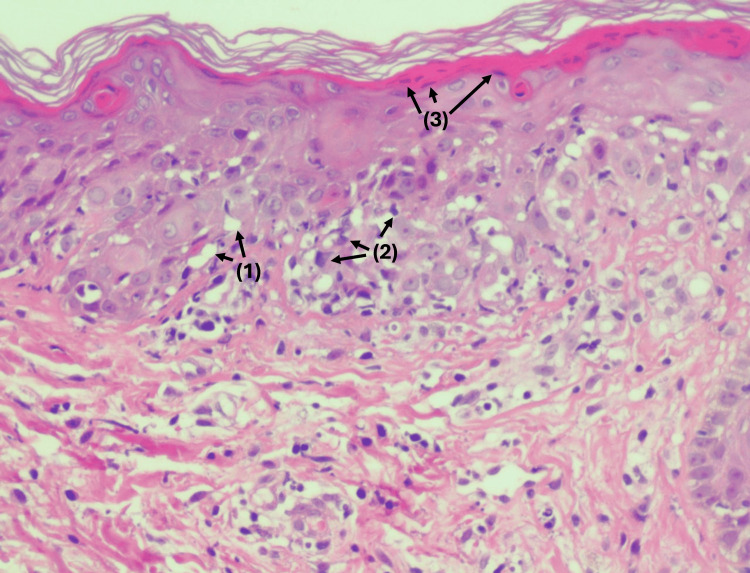
Skin biopsy of the patient with diffuse erythema of the abdomen and trunk showing focal full-thickness epidermal necrosis with interface dermatitis. Key histopathological features, including (1) basal cell vacuolar degeneration, (2) lymphocytic infiltrate at the dermo-epidermal junction, and (3) epidermal necrosis, are marked in the image for clarity. Epidermal necrosis appears as shrunken, eosinophilic (pink-staining) keratinocytes with pyknotic nuclei (condensed, dark nuclei) and a loss of cellular detail, indicating cell death.

Skin biopsy sections reveal focal full-thickness epidermal necrosis with prominent interface dermatitis, characterized by vacuolar degeneration of the basal cell layer and scattered necrotic keratinocytes throughout the epidermis. Areas of subepidermal cleft formation are identified. At the dermo-epidermal junction, there is a band-like lymphocytic infiltrate, with associated papillary dermal edema and perivascular lymphocytic aggregates, occasionally admixed with eosinophils. Importantly, there is no evidence of leukocytoclasia, microabscess formation, or festooning of dermal papillae (Figure 5). The interpretation concluded that the histopathological features are most consistent with interface dermatitis in the setting of ACLE (TEN-like variant/RS), correlating with the clinical and serological findings (Table 2).

**Table 2:** Summary of key clinical and diagnostic findings. TEN, toxic epidermal necrolysis; ACLE, acute cutaneous lupus erythematosus

Domain	Key Findings	Diagnostic Relevance
Clinical	Diffuse erythematous to violaceous plaques with epidermal detachment and mucosal involvement	Consistent with TEN-like ACLE
Serology	ANA, anti-dsDNA, anti-Sm, and anti-Ro/SSA positive; low complement (C3, C4)	Confirms active lupus autoimmunity
Histopathology	Full-thickness epidermal necrosis with interface dermatitis and dermal lymphocytic infiltrate	Characteristic of TEN-like lupus; rules out drug-induced TEN
Drug exposure	No recent high-risk medications identified	Supports autoimmune, not drug-related etiology
Systemic involvement	Mild renal and hepatic impairment; no major visceral involvement	Suggests primarily cutaneous lupus flare

Management and outcome

The patient was managed collaboratively by the rheumatology team, with a working diagnosis of a rare manifestation of SLE presenting as TEN-like ACLE with clinical features consistent with RS.

The rheumatology team initiated treatment with pulse therapy using intravenous methylprednisolone 1 g daily for five days, followed by oral prednisone 60 mg daily for one month, with a tapering schedule of 5 mg every two weeks. The patient was also started on trimethoprim-sulfamethoxazole (960 mg) orally three times per week for prophylaxis. Intravenous immunoglobulin (IVIG) was commenced at a dose of 1 g/kg administered over two consecutive days, in accordance with hospital protocol. Additionally, hydroxychloroquine 200 mg daily was prescribed. To provide supportive care, the patient received omeprazole for gastric protection, along with calcium and vitamin D supplementation to prevent corticosteroid-induced bone loss, and trimethoprim-sulfamethoxazole prophylaxis to reduce the risk of opportunistic infections.

Given the presence of vascular involvement, nifedipine and sildenafil were administered to improve peripheral circulation. For cutaneous management, topical corticosteroids, including betamethasone cream, were applied to active skin lesions to reduce inflammation, and clobetasol ointment was applied to the soles of the feet, while mupirocin ointment was used to prevent secondary bacterial infection. Additionally, lubricating eye drops (Optive) were prescribed to alleviate ocular dryness and protect the corneal surface.

The patient responded favorably to the instituted treatment, showing marked improvement in cutaneous and systemic symptoms over the ensuing weeks, with no evidence of new lesion development or systemic flare during follow-up.

## Discussion

TEN-like ACLE is an uncommon yet severe dermatologic manifestation of SLE, often posing a diagnostic and therapeutic challenge due to its clinical similarity to drug-induced TEN. This case highlights a rare presentation of SLE manifesting with extensive epidermal detachment mimicking TEN, coexisting with features of RS.

TEN-like ACLE: clinical and pathological characteristics

TEN-like ACLE is characterized by widespread epidermal necrosis, erythematous maculopapular lesions, and vesiculobullous eruptions predominantly on sun-exposed areas, often accompanied by mucosal involvement and systemic lupus serologies [2,4]. Unlike classic drug-induced TEN, TEN-like ACLE tends to develop gradually over days to weeks rather than acutely and is typically associated with positive ANA and lupus-specific autoantibodies [3,8]. Histopathology in such cases demonstrates full-thickness epidermal necrosis, interface dermatitis, and basal layer vacuolar changes, as seen in our patient, rather than the typical subepidermal blistering of drug-induced TEN [2,4].

Several authors emphasize that distinguishing TEN-like ACLE from drug-induced TEN is crucial, as the latter demands immediate drug withdrawal and supportive care, while TEN-like ACLE requires aggressive immunosuppressive therapy to control systemic lupus activity [2,5]. In our case, the patient was initially managed with high-dose corticosteroids and mycophenolate mofetil, which aligns with the current therapeutic recommendations for severe ACLE [1].

Rowell syndrome: an overlapping phenomenon

RS, first described by Rowell et al. in 1963, refers to the coexistence of lupus erythematosus and erythema multiforme (EM)-like lesions with immunological features such as positive ANA and anti-Ro/La antibodies [6,7]. Although historically debated, recent literature supports RS as a distinct clinical entity, often associated with a positive speckled ANA pattern, anti-Ro/SSA, anti-La/SSB antibodies, and sometimes anti-Sm antibodies [6,9]. Our patient did not fulfill the updated diagnostic criteria for RS [10], as the direct immunofluorescence of the skin biopsy was negative. Also, the targetoid-like lesion (annular) and the less severe form of the disease (multi-organ affection) are absent so the diagnosis is far from Rowell syndrome, despite the presence of EM-like lesions that go with the diagnosis of TEN-like ACLE.

Organ involvement and risk of multiorgan damage

In TEN-like ACLE, the most commonly affected extracutaneous organ is the kidney, reflecting active lupus nephritis that often accompanies severe cutaneous flares. Additional systemic involvement may include hematologic abnormalities (such as anemia and thrombocytopenia) and electrolyte disturbances like hyponatremia. By contrast, drug-induced TEN carries the highest risk of widespread multiorgan failure, involving hepatic, renal, and pulmonary systems, primarily due to the systemic inflammatory response and sepsis. RS, on the other hand, typically remains cutaneous with limited systemic impact, and severe multiorgan dysfunction is uncommon. Therefore, while all three conditions can present with extensive skin involvement, the prognosis and systemic risk differ significantly, with drug-induced TEN being the most life-threatening due to multiorgan failure, followed by TEN-like ACLE with lupus-related organ involvement, whereas RS generally demonstrates a more benign systemic profile.

Diagnostic challenges and clinical implications

The diagnostic challenge in such cases stems from the overlapping features between TEN-like ACLE, drug reactions, and RS. Table 3 summarizes key distinguishing features among these entities.

**Table 3:** Comparison of TEN-like ACLE, drug-induced TEN, and Rowell syndrome (RS). TEN, toxic epidermal necrolysis; ACLE, acute cutaneous lupus erythematosus; EM, erythema multiforme

Domain	TEN‑Like ACLE	Drug‑Induced TEN	Rowell Syndrome (RS)
Typical trigger	SLE flare; UV/photo‑exposure; intercurrent infection; no required culprit drug	High‑risk medication exposure (e.g., certain antibiotics, anticonvulsants, allopurinol) within a characteristic latency	LE flare with EM‑like morphology; may follow infection or photosensitivity
Tempo of onset	Subacute (days–weeks), often progressive	Acute, often rapid escalation after exposure	Subacute or relapsing course
Distribution	Frequently photo‑accentuated; may generalize	Generalized; not typically photo‑accentuated	Trunk and extremities; targetoid/EM‑like lesions
Primary morphology	Erythematous macules/patches → vesicles/bullae → epidermal detachment	Dusky macules → coalescence → sheet‑like epidermal slough	EM‑like lesions (targetoid), sometimes with LE features
Mucosal involvement	Variable; usually milder than drug‑TEN	Prominent and severe (oral, ocular, genital)	Uncommon/mild
Nikolsky sign	Often negative or focal	Often positive	Usually negative
Body surface area	Can be extensive; may reach >30%	Frequently >30% in severe cases	Typically limited
Systemic features	May accompany active SLE (fever, cytopenias, nephritis, hypocomplementemia)	Drug reaction‑related systemic illness, risk of multiorgan involvement	Features of LE: systemic illness less dramatic than drug‑TEN
Histopathology	Interface dermatitis with vacuolar change; full‑thickness keratinocyte necrosis; subepidermal clefts, and lymphocytic interface infiltrate	Widespread keratinocyte necrosis with scant interface change; apoptotic keratinocytes, and sparse inflammation	LE‑type interface dermatitis; necrotic keratinocytes typical of EM‑like lesions
Direct immunofluorescence (DIF)	Lupus band at DEJ (IgG/IgM/C3) often present	Negative or nonspecific	LE‑pattern immune deposits (lupus band) may be present
Serology	ANA positive; may have anti‑dsDNA, anti‑Sm, anti‑RNP; low C3/C4 common	Autoimmune serology typically absent; there is no hypocomplementemia pattern	Speckled ANA common; anti‑Ro/SSA ± anti‑La/SSB; RF may be positive
Culprit drug relationship	None required; many cases without a plausible drug	Necessary; clear temporal association and improvement after withdrawal	Not drug‑driven, but rather an LE variant with EM‑like lesions
Recurrence	Possible with SLE flares/UV	Recurs with re‑exposure	May relapse with LE activity
First‑line management	High‑dose systemic corticosteroids; immunosuppressants (e.g., mycophenolate, cyclophosphamide); strict photoprotection; supportive care	Immediate drug withdrawal, intensive supportive care (burn‑unit principles); consider IVIG/other adjuncts per local protocols	Systemic corticosteroids ± steroid‑sparing agents; antimalarials, and photoprotection
Prognosis	Generally better than drug‑TEN with timely immunosuppression; guided by SLE control	High morbidity; risk of serious complications	Usually favorable with LE‑directed therapy
Key pitfalls	Mislabeling as drug‑TEN → delay in immunosuppression	Misdiagnosing lupus flare as drug‑TEN can alter therapy; confirm drug timeline	Overdiagnosis without EM‑like lesions or RS‑profile serology

Our patient had a recent history of fluconazole use, which could have confounded the clinical picture, as fluconazole is rarely implicated in SJS/TEN. However, the gradual progression of lesions, absence of a positive Nikolsky sign, histological findings, and strong serological evidence of SLE favored TEN-like ACLE rather than drug-induced TEN.

Moreover, hyponatremia, hypoalbuminemia, and renal impairment in our case suggested active systemic lupus nephritis, emphasizing the systemic involvement that often accompanies severe cutaneous flares [11].

Management and outcomes

The cornerstone of management in TEN-like ACLE involves high-dose corticosteroids, immunosuppressants (such as mycophenolate mofetil and cyclophosphamide), and antimalarials [1,9]. Our patient responded favorably to systemic corticosteroids and mycophenolate, consistent with prior reports [2,5]. Supportive care with infection prophylaxis, gastric protection, and vascular support was essential to prevent complications.

Review

A review of published cases indicates that TEN-like ACLE is extremely rare, with fewer than 50 cases reported worldwide [3,4]. Similarly, RS remains a rare and debated entity, though newer literature highlights its autoimmune basis and overlaps with ACLE [6,10]. The similarity of clinical (dermatological) manifestations of both conditions exceedingly underscores the need for heightened clinical suspicion and multidisciplinary management. Table 4 summarizes previously reported cases of TEN-like ACLE and RS, along with the present case, to highlight diagnostic heterogeneity and management outcomes.

**Table 4:** Reported cases of TEN-like ACLE and Rowell syndrome (RS), including the present case. TEN, toxic epidermal necrolysis; ACLE, acute cutaneous lupus erythematosus; CLE, cutaneous lupus erythematosus; DIF, direct immunofluorescence

Author (Year)	Clinical Phenotype	Patients (Design)	Key Cutaneous Features	Immunology relevant to RS	Histology/DIF	Treatment/Outcome
Roberts et al., 2021 [2]	TEN-like lupus (review)	Review	Epidermal loss in acute severe SLE; no suitable culprit drug	Autoimmune serologies typical for SLE; RS not central	CLE-pattern histology/DIF	Systemic steroids ± adjuncts; supportive care.
Zargham et al., 2020 [3]	TEN-like ACLE as the first SLE manifestation	1 adult (case)	TEN-like erosive eruption; no culprit drug	ANA+; SLE serologies; RS panel not emphasized	Compatible with TEN-like CLE	Systemic steroids; recovery.
Romero et al., 2018 [4]	TEN-like ACLE	1 adult (case + literature review)	Widespread denudation/blistering mimicking TEN	ANA+, anti-dsDNA; RS profile not detailed	Interface dermatitis with full-thickness epidermal necrosis; lupus band possible	Systemic corticosteroids improved.
Kamble et al., 2025 [5]	ACLE mimicking TEN (TEN-like ACLE)	1 adult (case)	TEN-like rash in known SLE	SLE serologies; RS panel not fulfilled	CLE histology	Pulse methylprednisolone → improvement.
Khayyat et al., 2025 [6]	Rowell syndrome (review)	Narrative review	EM-like morphology in LE	Summarizes RS serologic criteria and pathogenesis	–	Therapeutic horizon overview.
Rowell et al., 1963 [7]	Rowell syndrome (original description)	Multiple cases	LE + EM-like lesions (not TEN-like)	Speckled ANA, RF+, anti-Ro/La	Interface changes of LE	Corticosteroids (era-appropriate).
Abdelmouttalib et al., 2021 [8]	TEN-like ACLE	2 women with SLE (case series)	Photo-accentuated TEN-like sloughing (BSA >30%)	SLE serologies positive; RS profile not fulfilled	Lupus band positive; CLE histology	High-dose corticosteroids → healing in ~2 weeks.
Gallo et al., 2020 [9]	Rowell syndrome (diagnostic challenge)	1 adult (case)	EM-like lesions with LE	RS triad fulfilled (speckled ANA; anti-Ro, and RF)	Interface dermatitis	Corticosteroids ± steroid-sparing agent.
Müller et al., 2011 [10]	Rowell syndrome (case + diagnostic accuracy)	1 adult (case report)	LE with EM-like lesions	Speckled ANA; anti-Ro/La/RF as per criteria	Interface dermatitis of LE	Corticosteroids; improvement.
Sethy et al., 2021 [12]	Rowell Syndrome	Single case	EM-like rash with SLE	ANA speckled, anti-Ro+	Interface dermatitis	Corticosteroids, hydroxychloroquine → remission
Bhattarai et al., 2024 [13]	Rowell Syndrome	Single case	EM-like lesions	ANA speckled, anti-Ro+	Interface dermatitis	Corticosteroids, immunosuppressants → improved
Wu et al., 2020 [14]	Rowell Syndrome + Lupus Hepatitis	Single case	EM-like lesions, liver involvement	ANA+, anti-Ro+	Interface dermatitis	Corticosteroids, hepatoprotective → improved
Present case (2025)	TEN-like ACLE with RS-type serologies	1 adult (case)	Diffuse TEN-like denudation; photo-accentuation; Nikolsky negative, and mild mucosal crusting	ANA positive (speckled); anti-Ro60+++; anti-La+; anti-Sm+++; Sm/RNP+++; RF <20; low complements; anti-dsDNA high	Full-thickness epidermal necrosis with interface dermatitis; subepidermal clefting, and lymphocytic DEJ infiltrate	High-dose prednisolone + mycophenolate; adjuncts; clinical improvement.

## Conclusions

TEN-like ACLE is an uncommon and severe variant of SLE, generally presenting with massive epidermal detachment, erosions, and mucocutaneous involvement, thereby clinically simulating drug-induced severe reactions seen in toxic epidermal necrolysis. It is important to differentiate the entities since treatment and prognostic outcomes differ drastically. Our case iterates the difficulty in diagnosis when TEN-like ACLE merges with the serological marker constellation of RS, including speckled ANA pattern and anti-Ro/SSA and anti-La/SSB positivity as well as drug-induced TEN. This issue raises the pertinent question of whether such entities constitute separate syndromes or phenotypic expressions along the lupus spectrum.

The clinician must consider early diagnosis of life-threatening complications to ensure timely immunosuppressive treatment, which remains the mainstay of management. Our case showed good results with systemic corticosteroids in high doses and mycophenolate mofetil, along with measures supportive to the patient's general condition, such as infection prophylaxis and vascular support. However, because of the rarity of cases reported in the literature, treatment still remains nonstandardized, and therapeutic management is, therefore, largely empirical. The case hence adds to the growing evidence that may depict an immunopathological continuum between TEN-like ACLE and RS. This case underscores the diagnostic lesson that early recognition and differentiation of TEN-like lupus variants are crucial for timely immunosuppressive therapy and improved outcomes. Furthermore, continued reporting and future research are essential to refine disease classification and establish standardized treatment protocols. Further studies, including detailed immunologic and genetic work-ups, will be required to best explore such mechanisms, narrow the diagnostic criteria, and engineer consensus-based management strategies. Any clinician should maintain a high index of suspicion concerning lupus emergencies with dermatologic manifestations, especially in cases exhibiting diffuse cutaneous necrosis without antecedent history of exposure to high-risk drugs.
